# Supplementary material for: Species-Wide Phylogenomics of the Staphylococcus aureus Agr Operon Revealed Convergent Evolution of Frameshift Mutations
Source: Microbiol Spectr. 2022 Jan 19;10(1):e01334-21. doi: 10.1128/spectrum.01334-21 (PMC8768832; doi:10.1128/spectrum.01334-21)
Supplement: SUPPLEMENTAL FILE 2 — Supplemental material. Download SPECTRUM01334-21_Supp_1_seq4.pdf, PDF file, 0.4 MB [file spectrum01334-21_supp_1_seq4.pdf]

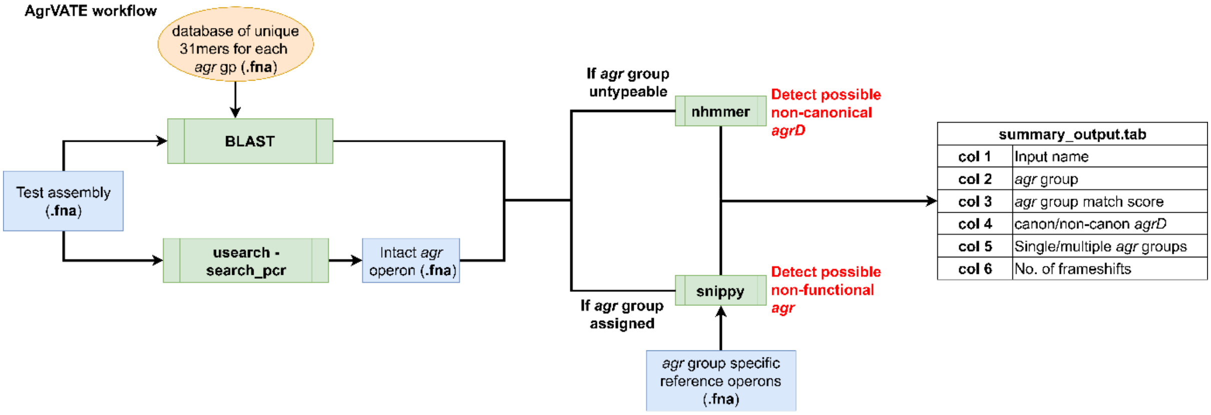


**Fig S1**: The AgrVATE pipeline is described for a single input (fasta file) genome assembly of *S. aureus*. Briefly, AgrVATE accepts a *S. aureus* genome assembly as input and performs a kmer search using an *agr* group-specific kmer database to assign the *agr* group. The *agr* operon is then extracted using i*n-silico* PCR and variants are called using an *agr* group specific reference operon. If the *agr* group is untypeable by AgrVATE, nhmmer search is performed to detect the presence of *agrD*, after which *ad hoc* bioinformatic steps are performed to identify the *agr* group.

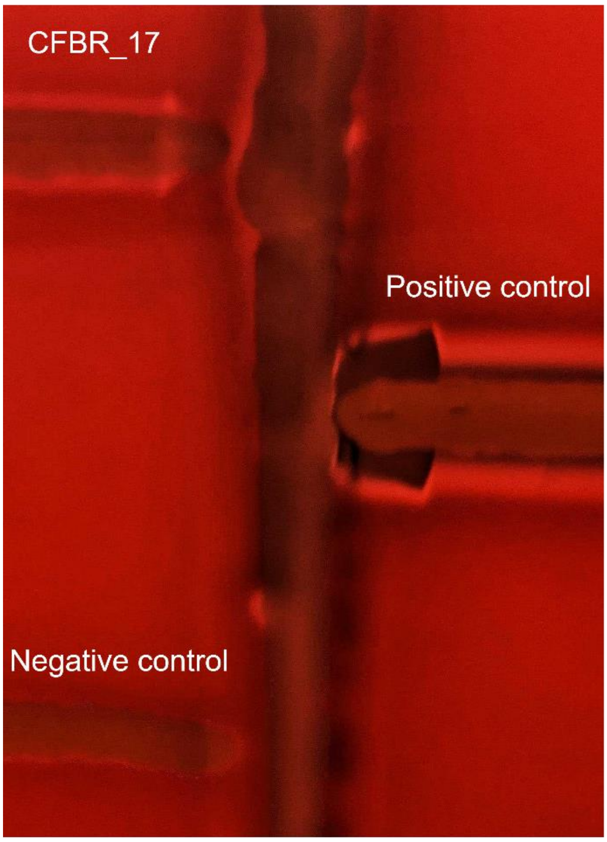


**Fig S2: Strain CFBR_17** shows weak haemolysis activity on Sheep Blood Agar plate. CAMP test as described in Traber et al, 2008 (Traber KE, Lee E, Benson S, Corrigan R, Cantera M, Shopsin B, Novick RP. Microbiology 2008;154(Pt 8):2265-74. doi: 10.1099/mic.0.2007/011874-0.) was performed to verify the haemolysis status of strain CFBR_17. USA300 JE2 strain was used as a haemolysis positive control, strain RN4220 was used as the vertical haemolysis negative strain and strain CFBR_15, a previously observed haemolysis negative strain (Bernardy EE, Petit RA, 3rd, Raghuram V, Alexander AM, Read TD, Goldberg JB. mBio. 2020;11(3). doi: 10.1128/mBio.00735-20), was used as a secondary negative control.

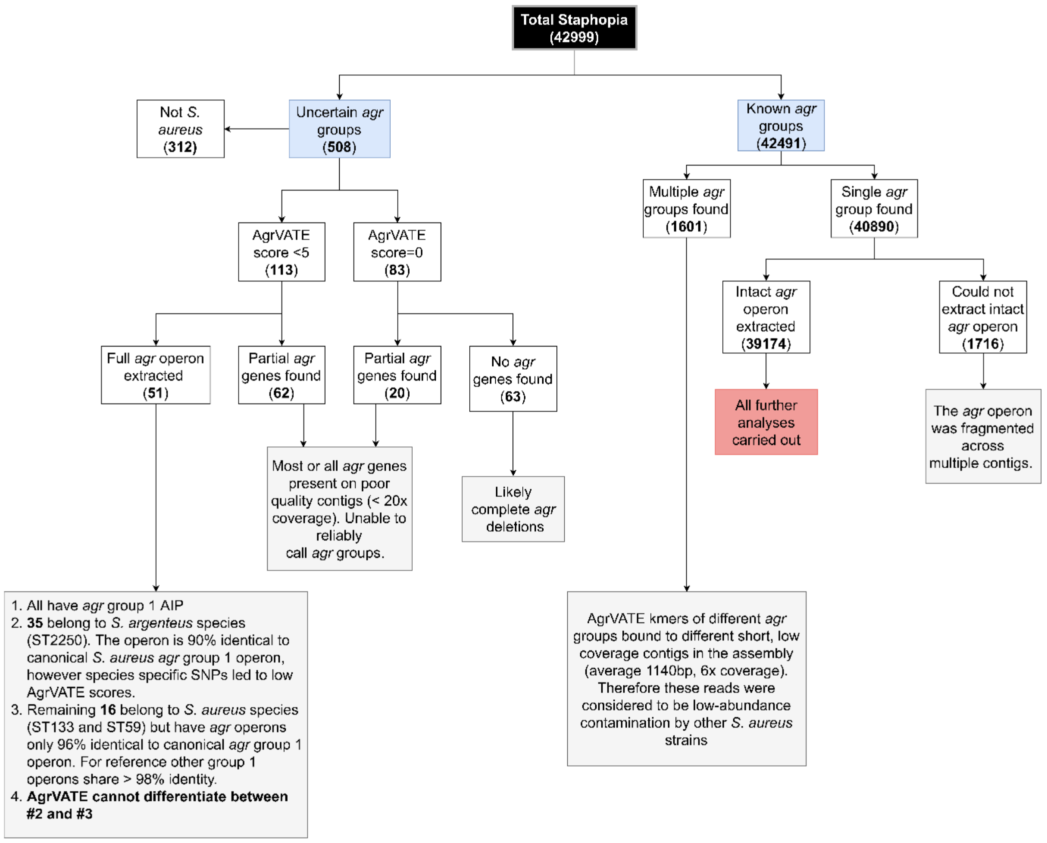


**Fig S3**: ***agr* typing status of 42,999 genomes from the Staphopia database.** All possible outcomes from AgrVATE were analysed further to determine reason(s) behind failed/uncertain *agr* group assignments. BLAST searches for individual *agr* genes and AIP sequences were performed to identify presence of *agr* genes. Contig length and coverage information were obtained from fasta header lines from SPAdes assemblies which are a part of the Staphopia analysis pipeline (Bankevich A, Nurk S, Antipov D, Gurevich AA, Dvorkin M, Kulikov AS, Lesin VM, Nikolenko SI, Pham S, Prjibelski AD, Pyshkin AV, Sirotkin AV, Vyahhi N, Tesler G, Alekseyev MA, Pevzner PA. J Comput Biol. 2012;19(5):455-77. doi: 10.1089/cmb.2012.0021) (Petit RA, 3rd, Read TD. PeerJ. 2018;6:e5261. doi: 10.7717/peerj.5261.) Accession AP018562 was used as a reference for *S. argenteus* ST2250 strains (Thaipadungpanit J, Amornchai P, Nickerson EK, Wongsuvan G, Wuthiekanun V, Limmathurotsakul D, Peacock SJ. J Clin Microbiol. 2015;53(3):1005-8. doi: 10.1128/JCM.03049-14)

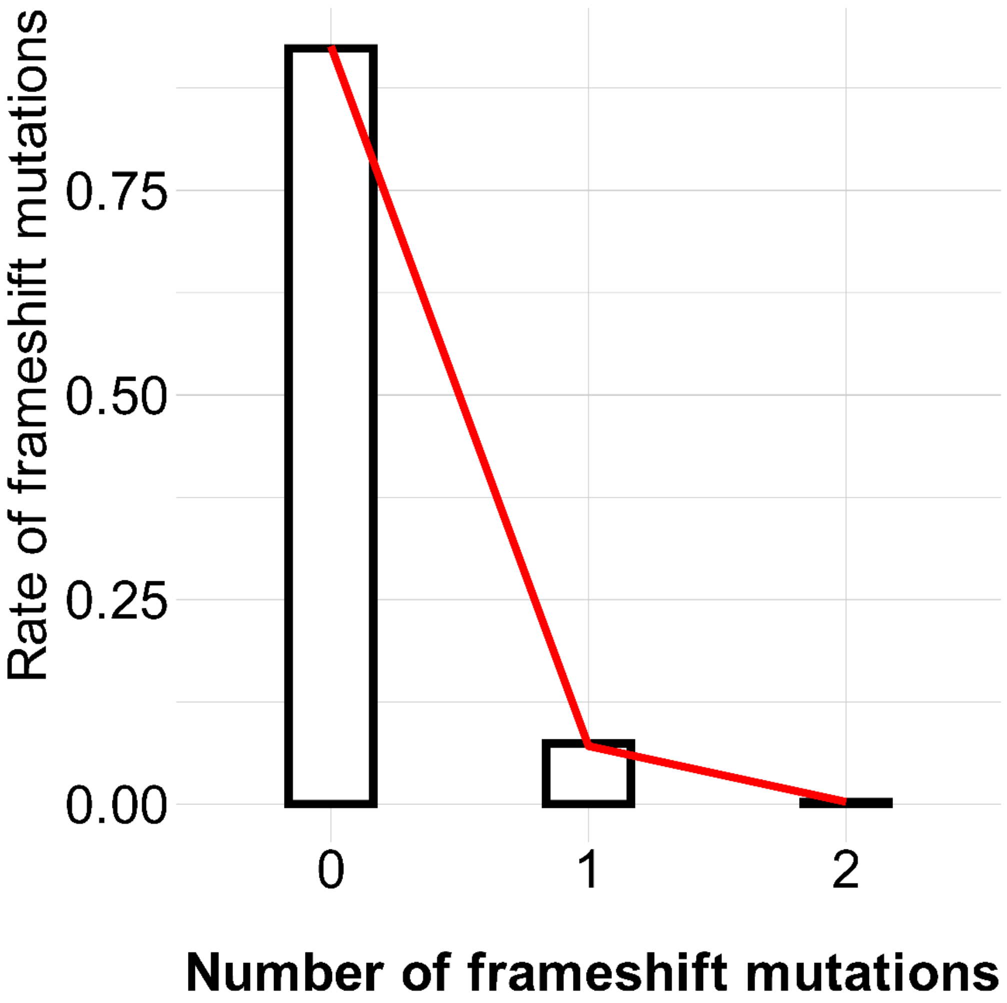


**Fig S4**: **Observed and expected frameshift mutation rates of the *agr* operon.** Bars show observed frameshift rates in the *agr* operon and red line shows the frameshift rate as modelled by a Poisson distribution ($\lambda$= 0.07650482462) From a total of 39,174 *agr* operons, 36177 operons had zero frameshifts, 2906 had one frameshift and 91 had two frameshifts. Poisson distribution was modelled in R using the dpois() function.

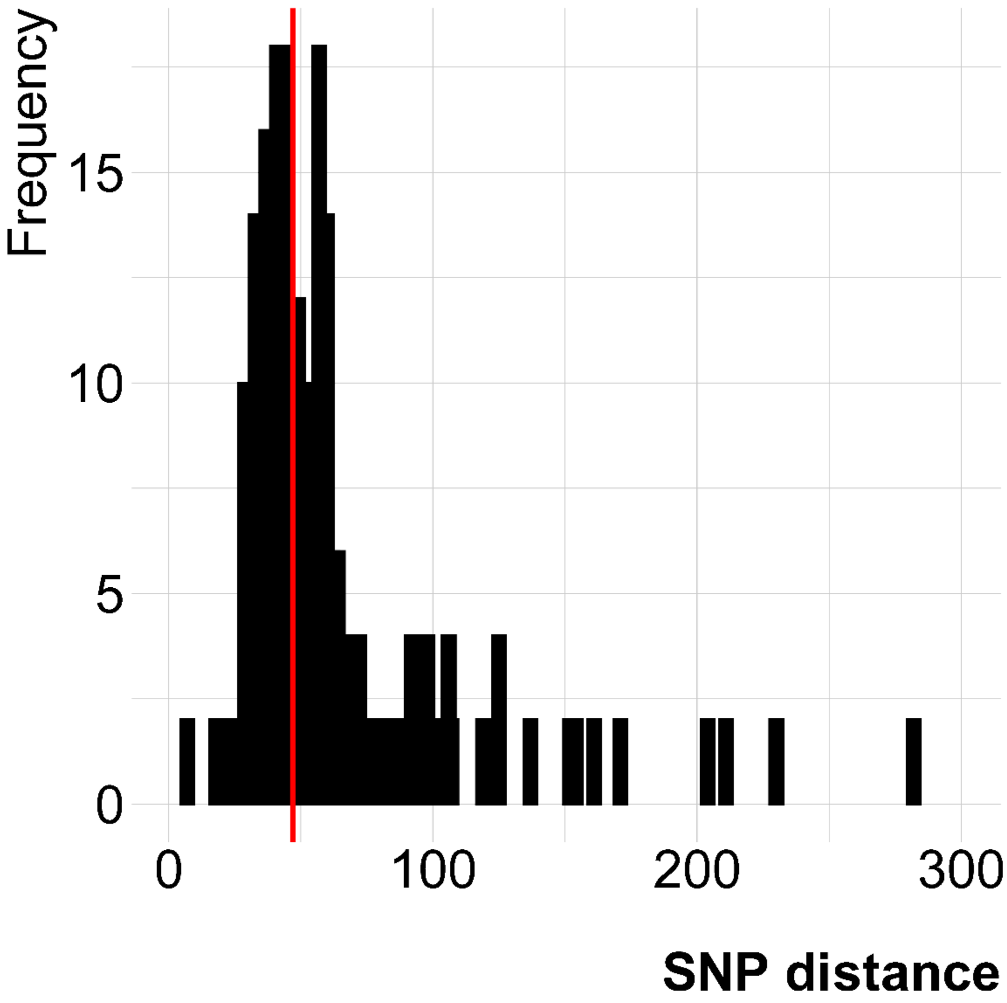


**Fig S5: Pairwise SNP distance distribution for Staphopia database NRD set (380 genomes) where pairwise Mash distances were < 0.0005**. Bars represent frequency of pairs having Mash distance < 0.0005. A mash distance cut-off of 0.0005 represents strains that are at maximum 282 SNPs apart, with a median SNP distance of 47 (red line).

| Biosample | Strain ID | Patient ID | *agr* group | Match score | No. of *agr* | Frameshift | sheep blood haemolysis |
|---|---|---|---|---|---|---|---|
| SAMN09847804 | BCH-SA-01 | 1 | 2 | 15 | s | 0 | - |
| SAMN09847805 | BCH-SA-02 | 2 | 2 | 15 | s | 1 | - |
| SAMN09847806 | BCH-SA-03 | 3 | 1 | 13 | s | 0 | + |
| SAMN09847807 | BCH-SA-04 | 4 | 3 | 15 | s | 0 | + |
| SAMN09847808 | BCH-SA-05 | 5 | 2 | 15 | s | 0 | + |
| SAMN09847809 | BCH-SA-06 | 5 | 2 | 15 | s | u | + |
| SAMN09847810 | BCH-SA-07 | 6 | 1 | 13 | s | 0 | + |
| SAMN09847811 | BCH-SA-08 | 7 | 3 | 15 | s | u | + |
| SAMN09847812 | BCH-SA-09 | 8 | 1 | 13 | s | 0 | + |
| SAMN09847813 | BCH-SA-10 | 9 | 1 | 13 | s | u | - |
| SAMN09847814 | BCH-SA-11 | 10 | 3 | 15 | s | 0 | - |
| SAMN09847815 | BCH-SA-12 | 11 | 2 | 15 | s | 0 | + |
| SAMN09847816 | BCH-SA-13 | 12 | 1 | 13 | s | 0 | + |
| SAMN09847817 | BCH-SA-14 | 13 | 1 | 13 | s | 0 | + |
| SAMN09847818 | BCH-SA-15 | 14 | 1 | 13 | s | 0 | + |
| SAMN09847832 | CFBR_01 | CFBR-122 | 2 | 14 | s | 0 | + |
| SAMN09847836 | CFBR_02 | CFBR-148 | 2 | 15 | s | 0 | - |
| SAMN09847841 | CFBR_03 | CFBR-153 | 1 | 13 | s | 1 | - |
| SAMN09847844 | CFBR_04 | CFBR-172 | 3 | 15 | s | 0 | - |
| SAMN09847850 | CFBR_05 | CFBR-238 | 2 | 15 | s | 0 | + |
| SAMN09847840 | CFBR_06 | CFBR-152 | 2 | 15 | s | 0 | - |
| SAMN09847842 | CFBR_07 | CFBR-170 | 2 | 15 | s | 0 | - |
| SAMN09847846 | CFBR_08 | CFBR-196 | 2 | 15 | s | 1 | - |
| SAMN09847849 | CFBR_09 | CFBR-219 | 2 | 15 | s | 0 | + |
| SAMN09847834 | CFBR_10 | CFBR-134 | 2 | 15 | s | 0 | + |
| SAMN09847823 | CFBR_11 | CFBR-101 | 2 | 15 | s | 0 | - |
| SAMN09847845 | CFBR_12 | CFBR-178 | 2 | 15 | s | 0 | - |
| SAMN09847848 | CFBR_13 | CFBR-213 | 1 | 13 | s | 0 | + |
| SAMN09847856 | CFBR_14 | CFBR-316 | 1 | 13 | s | 1 | - |
| SAMN09847835 | CFBR_15 | CFBR-146 | 2 | 15 | s | 1 | - |
| SAMN09847830 | CFBR_16 | CFBR-105 | 2 | 15 | s | 0 | + |
| SAMN09847824 | CFBR_17 | CFBR-102 | 2 | 15 | s | 1 | + |
| SAMN09847831 | CFBR_18 | CFBR-120 | 1 | 13 | s | 0 | + |
| SAMN09847833 | CFBR_19 | CFBR-123 | 2 | 15 | s | 0 | + |
| SAMN09847837 | CFBR_20 | CFBR-149 | 2 | 14 | s | 0 | + |
| SAMN09847838 | CFBR_21 | CFBR-150 | 2 | 15 | s | 2 | - |
| SAMN09847839 | CFBR_22 | CFBR-150 | 2 | 15 | s | 2 | - |
| SAMN09847843 | CFBR_23 | CFBR-171 | 2 | 15 | s | 0 | + |
| SAMN09847847 | CFBR_24 | CFBR-201 | 2 | 15 | s | 0 | + |
| SAMN09847819 | CFBR_25 | CFBR-101 | 2 | 15 | s | 0 | + |
| SAMN09847820 | CFBR_26 | CFBR-101 | 2 | 15 | s | 0 | + |
| SAMN09847821 | CFBR_27 | CFBR-101 | 1 | 13 | m | 0 | + |
| SAMN09847822 | CFBR_28 | CFBR-101 | 2 | 15 | s | 0 | + |
| SAMN09847825 | CFBR_29 | CFBR-105 | 2 | 15 | s | 0 | + |
| SAMN09847826 | CFBR_30 | CFBR-105 | 2 | 15 | s | 0 | + |
| SAMN09847827 | CFBR_31 | CFBR-105 | 2 | 15 | s | 0 | + |
| SAMN09847828 | CFBR_32 | CFBR-105 | 2 | 15 | s | 0 | + |
| SAMN09847829 | CFBR_33 | CFBR-105 | 2 | 15 | s | 0 | + |
| SAMN09847851 | CFBR_34 | CFBR-280 | 2 | 15 | s | 0 | - |
| SAMN09847852 | CFBR_35 | CFBR-280 | 1 | 13 | s | 0 | + |
| SAMN09847853 | CFBR_36 | CFBR-280 | 2 | 15 | s | 0 | - |
| SAMN09847854 | CFBR_37 | CFBR-309 | 3 | 15 | s | 0 | + |
| SAMN09847855 | CFBR_38 | CFBR-314 | 1 | 13 | s | 0 | + |
| SAMN09847857 | CFBR_39 | CFBR-322 | 3 | 15 | s | u | + |
| SAMN09847858 | CFBR_40 | CFBR-336 | 2 | 15 | s | 0 | + |

| Biosample | Strain ID | Patient ID | *agr* group | Match score | No. of *agr* | Frameshift | sheep blood haemolysis |
|---|---|---|---|---|---|---|---|
| SAMN09847859 | CFBR_41 | CFBR-429 | 1 | 13 | s | 1 | - |
| SAMN09847860 | CFBR_42 | CFBR-430 | 1 | 12 | s | 0 | + |
| SAMN09847861 | CFBR_43 | CFBR-447 | 1 | 13 | s | 0 | + |
| SAMN09847862 | CFBR_44 | CFBR-487 | 1 | 13 | s | 0 | + |
| SAMN09847863 | CFBR_45 | CFBR-487 | 1 | 13 | s | 0 | + |
| SAMN09847864 | CFBR_46 | CFBR-509 | 1 | 13 | s | 0 | + |
| SAMN09847865 | CFBR_47 | CFBR-515 | 1 | 13 | s | 0 | + |
| SAMN09847866 | CFBR_48 | CFBR-530 | 1 | 13 | s | 0 | + |
| SAMN09847867 | CFBR_49 | CFBR-573 | 1 | 13 | s | 0 | + |
| SAMN20708268 | CFBR623_Sau_20200220_P_90 | CFBR-623 | 1 | 13 | s | 0 | + |
| SAMN20708269 | CFBR623_Sau_20200220_P_91 | CFBR-623 | 1 | 13 | s | 0 | + |
| SAMN20708270 | CFBR623_Sau_20200220_S_92 | CFBR-623 | 1 | 13 | s | 0 | + |
| SAMN20708271 | CFBR623_Sau_20200220_S_93 | CFBR-623 | 1 | 13 | s | 0 | + |
| SAMN20708272 | CFBR623_Sau_20200220_S_94 | CFBR-623 | 1 | 13 | s | 0 | + |
| SAMN20708273 | CFBR623_Sau_20200220_S_95 | CFBR-623 | 1 | 13 | s | 0 | + |
| SAMN20708274 | CFBR623_Sau_20200220_S_96 | CFBR-623 | 1 | 13 | s | 0 | + |
| SAMN20708275 | CFBR623_Sau_20200220_S_97 | CFBR-623 | 1 | 13 | s | 0 | + |
| SAMN20708276 | CFBR196_Sau_20200312_P_100 | CFBR-196 | 2 | 15 | s | 0 | + |
| SAMN20708277 | CFBR196_Sau_20200312_S_101 | CFBR-196 | 2 | 15 | s | 0 | + |
| SAMN20708278 | CFBR196_Sau_20200312_S_102 | CFBR-196 | 2 | 15 | s | 0 | + |
| SAMN20708279 | CFBR196_Sau_20200312_S_103 | CFBR-196 | 2 | 15 | s | 0 | + |
| SAMN20708280 | CFBR196_Sau_20200312_S_104 | CFBR-196 | 2 | 15 | s | 0 | + |
| SAMN20708281 | CFBR196_Sau_20200312_P_105 | CFBR-196 | 2 | 15 | s | 0 | + |
| SAMN20708282 | CFBR196_Sau_20200312_S_106 | CFBR-196 | 2 | 15 | s | 0 | + |
| SAMN20708283 | CFBR196_Sau_20200312_S_107 | CFBR-196 | 2 | 15 | s | 0 | + |
| SAMN20708284 | CFBR196_Sau_20200312_S_108 | CFBR-196 | 2 | 15 | s | 0 | + |
| SAMN20708285 | CFBR196_Sau_20200312_S_109 | CFBR-196 | 2 | 15 | s | 0 | + |
| SAMN20708286 | CFBR311_Sau_20200312_P_110 | CFBR-311 | 2 | 15 | m | u | + |
| SAMN20708287 | CFBR311_Sau_20200312_S_111 | CFBR-311 | 2 | 15 | s | 1 | - |
| SAMN20708288 | CFBR311_Sau_20200312_S_112 | CFBR-311 | 2 | 15 | s | 1 | - |
| SAMN20708289 | CFBR311_Sau_20200312_S_113 | CFBR-311 | 2 | 15 | s | 1 | - |
| SAMN20708290 | CFBR311_Sau_20200312_S_114 | CFBR-311 | 2 | 15 | s | 1 | - |
| SAMN20708291 | CFBR311_Sau_20200312_S_115 | CFBR-311 | 2 | 15 | s | 1 | - |
| SAMN20708292 | CFBR311_Sau_20200312_S_116 | CFBR-311 | 2 | 15 | s | 1 | - |
| SAMN20708293 | CFBR311_Sau_20200312_S_117 | CFBR-311 | 1 | 13 | s | 0 | + |
| SAMN20708294 | CFBR311_Sau_20200312_S_118 | CFBR-311 | 1 | 13 | s | 0 | + |

**Table S1: AgrVATE and sheep blood haemolysis results from 88 *S. aureus* samples isolated from CF patients**. **Columns 1** corresponds to the Biosample accession number. **Columns 2 and 3** correspond to strain identifier and patient identifier of each sample respectively. Columns 4-8 are outputs of AgrVATE (https://github.com/VishnuRaghuram94/AgrVATE#outputs) and are as follows: **Column 4**: *agr* group as designated by AgrVATE, **Column 5**: Match score of the designated *agr* group (can range from 0 to 15), **Column 6**: Number of *agr* groups found (s: single, m: multiple), **Column 7**: Presence/absence of frameshifts (1: present, 0: absent, u: unknown).

**Column 8** represents the haemolysis status on Sheep Blood Agar (+ : haemolysis positive, - : haemolysis negative).

| | GLM | RF | XGB | KNN |
|---|---|---|---|---|
| **Sensitivity** | 0.561 | 0.617 | 0.607 | 0.645 |
| **Specificity** | 0.513 | 0.572 | 0.555 | 0.516 |
| **Pos Pred Value** | 0.012 | 0.014 | 0.014 | 0.013 |
| **Neg Pred Value** | 0.991 | 0.993 | 0.993 | 0.993 |
| **Precision** | 0.012 | 0.014 | 0.014 | 0.013 |
| **Recall** | 0.561 | 0.617 | 0.607 | 0.645 |
| **F1** | 0.023 | 0.028 | 0.027 | 0.026 |
| **Prevalence** | 0.01 | 0.01 | 0.01 | 0.01 |
| **Detection Rate** | 0.006 | 0.006 | 0.006 | 0.006 |
| **Detection Prevalence** | 0.487 | 0.43 | 0.447 | 0.486 |
| **Balanced Accuracy** | 0.537 | 0.594 | 0.581 | 0.58 |

**Table S2**: **Classifiers used to predict presence/absence of *agr* frameshifts using *agr* group, clonal complex, host body site and host infection/colonization status**. GLM – General Linear Model, RF – Random Forest, XGB – Extreme Gradient Boost, KNN – K nearest neighbours. Models were trained using the R package caret with a randomly sampled dataset comprising 400 frameshift+ and 400 frameshift- samples. Model summary parameters were computed using the confusionMatrix() function.

## Supplemental references


1. Traber KE, Lee E, Benson S, Corrigan R, Cantera M, Shopsin B, Novick RP. agr function in clinical Staphylococcus aureus isolates. Microbiology (Reading). 2008;154(Pt 8):2265-74. Epub 2008/08/01. doi: 10.1099/mic.0.2007/011874-0. PubMed PMID: 18667559; PMCID: PMC4904715.

2. Bernardy EE, Petit RA, 3rd, Raghuram V, Alexander AM, Read TD, Goldberg JB. Genotypic and Phenotypic Diversity of Staphylococcus aureus Isolates from Cystic Fibrosis Patient Lung Infections and Their Interactions with Pseudomonas aeruginosa. mBio. 2020;11(3). Epub 2020/06/25. doi: 10.1128/mBio.00735-20. PubMed PMID: 32576671; PMCID: PMC7315118.

3. Bankevich A, Nurk S, Antipov D, Gurevich AA, Dvorkin M, Kulikov AS, Lesin VM, Nikolenko SI, Pham S, Prjibelski AD, Pyshkin AV, Sirotkin AV, Vyahhi N, Tesler G, Alekseyev MA, Pevzner PA. SPAdes: a new genome assembly algorithm and its applications to single-cell sequencing. J Comput Biol. 2012;19(5):455-77. Epub 2012/04/18. doi: 10.1089/cmb.2012.0021. PubMed PMID: 22506599; PMCID: PMC3342519.

4. Petit RA, 3rd, Read TD. Staphylococcus aureus viewed from the perspective of 40,000+ genomes. PeerJ. 2018;6:e5261. Epub 2018/07/18. doi: 10.7717/peerj.5261. PubMed PMID: 30013858; PMCID: PMC6046195.

5. Thaipadungpanit J, Amornchai P, Nickerson EK, Wongsuvan G, Wuthiekanun V, Limmathurotsakul D, Peacock SJ. Clinical and molecular epidemiology of Staphylococcus argenteus infections in Thailand. J Clin Microbiol. 2015;53(3):1005-8. Epub 2015/01/09. doi: 10.1128/JCM.03049-14. PubMed PMID: 25568440; PMCID: PMC4390622.
